# Diversity-Generating Machines: Genetics of Bacterial Sugar-Coating

**DOI:** 10.1016/j.tim.2018.06.006

**Published:** 2018-12

**Authors:** Rafał J. Mostowy, Kathryn E. Holt

**Affiliations:** 1 Department of Infectious Disease Epidemiology, School of Public Health, Imperial College London, London, UK; 2 Department of Biochemistry and Molecular Biology, Bio21 Molecular Science and Biotechnology Institute, The University of Melbourne, Victoria, Australia; 3 The London School of Hygiene and Tropical Medicine, London, United Kingdom

**Keywords:** glycans, polysaccharide, recombination, ecology, vaccine, phage therapy

## Abstract

Bacterial pathogens and commensals are surrounded by diverse surface polysaccharides which include capsules and lipopolysaccharides. These carbohydrates play a vital role in bacterial ecology and interactions with the environment. Here, we review recent rapid advancements in this field, which have improved our understanding of the roles, structures, and genetics of bacterial polysaccharide antigens. Genetic loci encoding the biosynthesis of these antigens may have evolved as bacterial diversity-generating machines, driven by selection from a variety of forces, including host immunity, bacteriophages, and cell–cell interactions. We argue that the high adaptive potential of polysaccharide antigens should be taken into account in the design of polysaccharide-targeting medical interventions like conjugate vaccines and phage-based therapies.

## Highlights

Recent advances in glycobiology emphasise the importance of surface carbohydrates in the evolution of bacterial commensals and pathogens.

Epidemiological and genomic analyses highlight the remarkable diversity and adaptability of bacterial surface polysaccharides, often driven by horizontal DNA transfer.

Functional heterogeneity of bacterial polysaccharide antigens points to multiple factors which have shaped their diversity.

Antigenic diversity is a challenge for polysaccharide conjugate vaccines which strive to eliminate some bacterial diseases, like the pneumococcal disease.

## Diversity-Generating Machinery

The world of bacteria is extraordinarily diverse, and even the most superficial understanding of each species alone takes years of research. Therefore, even though most bacteria produce extracellular polysaccharides, the biology of polysaccharide production has more exceptions than rules. Nevertheless, a cross-sectional look at different bacterial species reveals many similar characteristics of carbohydrate antigens, and these similarities have important implications for their evolution. Here we compare the main aspects of polysaccharide antigen biology between different antibiotic-resistant global priority pathogens as defined by the World Health Organization in 2017 (www.who.int/mediacentre/news/releases/2017/bacteria-antibiotics-needed), particularly in relation to their functionality, genetics, and phenotypic variation.

## Functions of Polysaccharide Antigens and Their Role in Disease

Bacteria produce several extracellular polysaccharides which are crucial for their ability to colonise and cause disease. Gram-negative bacteria produce a lipopolysaccharide (LPS), which is an important component of the outer cell membrane and often includes a highly variable O-antigen at the end. Gram-negatives can also produce an additional capsular polysaccharide (CPS), which forms a surface layer. Gram-positive bacteria can synthesise teichoic acids, and like Gram-negatives, can also produce CPS, but they do not produce LPS. In addition to these major antigens, both Gram-negative and Gram-positive bacteria can synthesise exopolysaccharides (EPS), which are released into the environment and are not attached to the cell.

All of these extracellular polysaccharides play a variety of important roles in bacterial lifestyle and pathogenesis. Polysaccharide capsules enable bacterial evasion of the host immune system by shielding bacteria from the complement system, antibodies, or engulfment by macrophages [14]. Consequently, bacterial capsules are widely recognised as important virulence determinants 15, 16, 17, 18, 19, 20. A brief summary of major polysaccharide antigens in WHO priority pathogens is presented in Table 1. (Note that *Neisseria meningitidis* is not included as it does not appear on the WHO list; however, this is also an important encapsulated pathogen for which capsule-targeting vaccines are in use [21]). *Streptococcus pneumoniae* capsules facilitate escape from mucous entrapment, thus affecting bacterial ability to colonise and transmit 22, 23. The LPS, together with its external component, O-antigen, plays an important role in bacterial colonisation by protecting the cell from hydrophobic antibiotics [24], or inducing resistance against bacteriophages [25]. LPS and LOS (lipo-oligosaccharide, which is LPS without its external O-antigen) have both been shown to be virulence determinants in *Haemophilus, Neisseria, Campylobacter*, or Enterobacteriaceae 19, 21, 26, 27, 28, 29. Furthermore, both LPS and teichoic acid can contribute to adherence to epithelial cells 30, 31. Finally, the externally secreted EPS, like colanic acid in *Escherichia coli* or the alginate in *Pseudomonas aeruginosa*, forms the basis of biofilms – a self-produced polymeric matrix which enables bacterial adhesion to surfaces and protects colonies from environmental dangers, including desiccation, bacteriophages, antibiotics, or the host immune system [32].

**Table 1:** Comparison of Polysaccharide Antigens among WHO Priority Pathogens in Need of New Antibiotics^a^

Species	Class	Common habitat	Major PS antigens	Diversity	Biosynthesis	Refs
CRITICAL						
*Acinetobacter baumannii*	Gram−	Diverse environments	Capsule	38 serovars, 25 genetic clusters	wzy	[1]
*Pseudomonas aeruginosa*	Gram−	Diverse environments	O-antigens (2 types)	20 serogroups	wzy, ABC	2, 3
*Klebsiella pneumoniae*	Gram−	Diverse environments	Capsule, O-antigen	80 K-antigens, 8 O-antigens	wzy, ABC	[4]
*Escherichia coli*	Gram−	Animals	Capsule, O-antigen	180 O-antigens, 80 K-antigens	wzy, ABC	5, 6
HIGH						
*Enterococcus faecium*	Gram+	Animals	Teichoic acid, capsule	(Understudied)	Probably wzy	[7]
*Staphylococcus aureus*	Gram+	Animals	Capsule	11 serotypes	wzy	[8]
*Helicobacter pylori*	Gram−	Human stomach	O-antigen	At least eight types	wzk	[9]
*Campylobacter jejuni*	Gram−	Animals	Capsule, LOS	47 serotypes, LOS phase variation	ABC	[10]
*Salmonella enterica*^b^	Gram−	Diverse environments	O-antigen	46 O-antigens	wzy, ABC	[11]
*Neisseria gonorrhoeae*	Gram−	Humans	LOS	LOS phase variation	(none)	[12]
MEDIUM						
*Streptococcus pneumoniae*	Gram+	Human respiratory tract	Capsule, teichoic acid	∼100 serotypes	wzy, synth	[13]
*Haemophilus influenzae*	Gram−	Human respiratory tract	Capsule, LOS	Six serotypes, LOS phase variation	ABC	[12]

a Major polysaccharide antigens of WHO global priority pathogens in need of new antibiotics, divided into three categories according to the urgency of need for new antibiotics: critical, high and medium priority. In critical-priority Enterobacteriacae, only *K. pneumoniae* and *E. coli* are shown. PS, polysaccharide; LOS, lipo-oligosaccharide; wzy, wzy-dependent; synth, synthase-dependent.

b Typhi serovar also carries a capsule.

## Genetic Architecture and Evolution

**Figure 1:**
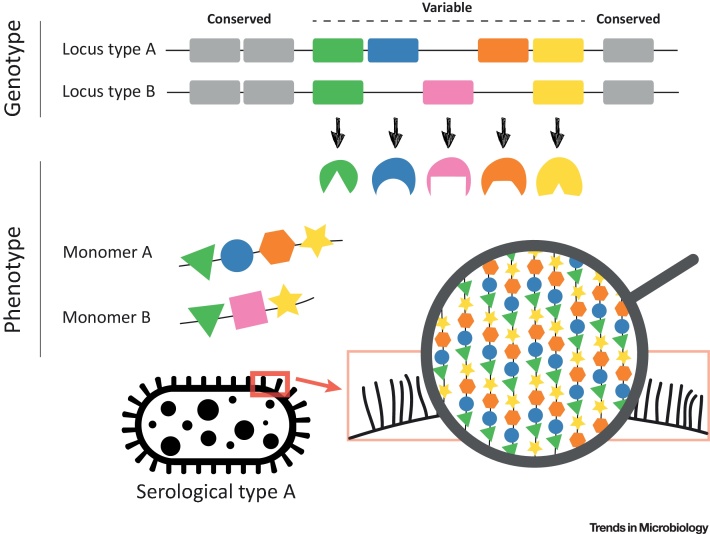
Generation of Polysaccharide Diversity in Bacteria. Polysaccharide antigens, like capsules and O-antigens, are usually synthesised by a specialist group of enzymes which are encoded by genes located in an antigen-biosynthesis locus. The genetic architecture of these loci is often similar between different, even distantly related, bacterial species. The specialised polymer-specific genes (coloured cassettes), which encode transferase enzymes (coloured shapes), are typically located in the middle of the locus. They are flanked by conserved, regulatory or transport genes (grey cassettes). The polymer-specific genes synthesise a monomer (so-called repeat unit), which is then polymerised to a polysaccharide chain and transported outside the cell. The order of these two events depends on the synthesis pathway, which, in the majority of studied cases, belongs to either the wzy-dependent or the ABC-dependent class. A given combination of the polymer-specific genes is a strong predictor of the polysaccharide structure, and thus bacterial serological type (serotype).

In spite of its length, a polysaccharide chain consists of a relatively small number of sugar molecules. The polymer can be biosynthesised in different ways, but in the large majority of cases it is done via one of three different mechanisms: the wzy-dependent, ABC-dependent, or synthase-dependent pathway (reviewed in 5, 33). These pathways engage sugar-specific enzymes to synthesise the polysaccharide, and the specific combination of these enzymes determines the sugar structure. There is a notable architectural similarity between genetic loci which synthesise polysaccharide chains (particularly wzy-synthesis and ABC-synthesis operons) in that the highly variable, polymer-specific region is located in the middle of the locus, and is surrounded by conserved genes that usually have roles in transport, assembly, export, or synthesis of sugars (in wzy-synthesised operons this also includes *wzx* and *wzy* genes). The typical design of a polysaccharide antigen locus in bacteria is shown in Figure 1, and its consequences for epidemiological serotyping are discussed in Box 1.

Box 1 **The Future of Serotyping**

Even though the sequence of genes located in the polysaccharide biosynthesis locus is highly predictive of the expressed polysaccharide, the genotype is not a perfect predictor of the phenotype for two main reasons. First, genetic mutations within those genes can alter specificity of enzymes encoded by them, thus altering a biochemical linkage and producing a new serotype. Second, additional genes located outside the synthesis locus can influence or direct the final sugar structure. This has important consequences for public health. With increasing adoption of high-throughput sequencing for strain characterisation by research and public health labs, *in silico* serotyping has now largely overtaken the standard serotyping methods, which require specialised reagents and expertise. (Table I summarises currently publicly available tools for *in silico* serotyping.) The flip side of this is that the gap between serologically-determined diversity and genetically-determined diversity is widening. Thus, biochemical characterisation of polysaccharide antigens remains important, and a good understanding of the complex genotype-phenotype map in polysaccharide antigens remains one of the great challenges of polysaccharide biology (see also Outstanding Questions).

Alt-text: Box 1

**Table I:** Public Tools for *In Silico* Serotyping Using Whole-Genome Data^a^

Name	Species	Data input	Precision	Website	Refs
PAst	*Pseudomonas aeruginosa*	SR or A	Entire locus	github.com/Sandramses/PAst	[102]
Kaptive	*Klebsiella pneumoniae*	A	Entire locus	kaptive.holtlab.net	[4]
SerotypeFinder	*Escherichia coli*	SR or A	*wzx/wzy/wzt/wzm*	cge.cbs.dtu.dk/services/SerotypeFinder	[103]
SRST2 + EcOH	*E. coli*	SR	*wzx/wzy/wzt/wzm*	github.com/katholt/srst2	[104]
SeqSero	*Salmonella*	SR or A	Entire locus	www.denglab.info/SeqSero	[105]
SISTR	*Salmonella*	A	Core genes	lfz.corefacility.ca/sistr-app	[106]
PneumoCat	*Streptococcus pneumoniae*	A	Entire locus	github.com/phe-bioinformatics/PneumoCaT	[107]
SeroBA	*S. pneumoniae*	SR	Entire locus	github.com/sanger-pathogens/seroba	[108]

a The list contains publicly available tools for *in silico* serotyping of WHO priority pathogens listed in Table 1. SR, short-reads; A, assembly.

The strikingly consistent architectural design of polysaccharide biosynthesis loci has important implications for the generation of antigenic diversity in bacteria. First, the synthesis of a monosaccharide via serotype-specific genes means that shuffling of these genes can alter the serotype. This can be achieved either via inactivation of one or more enzymes (gene loss), or an exchange leading to a new combination of enzymes (horizontal gene transfer) – often with the help of transposable elements – as has been widely observed in well studied *S. pneumoniae*, *E. coli*, or *Klebsiella pneumoniae* 4, 6, 34. Second, the presence of conserved genes at the flanking regions of the synthesis loci, like *dexB/aliA* in *S. pneumoniae* or *galF/gnd* in *Klebsiella*, promotes exchange of the entire locus by homologous recombination, which requires homology only at the flanks. Such changes facilitate serotype/antigen alterations between distant lineages without having to ‘invent’ a new combination, which has been widely documented in epidemiological studies 35, 36, 37, 38, 39, 40, 41, 42, 43. Third, genetic and epigenetic changes in regulatory genes (commonly referred to as ‘phase variation’) can affect capsule expression, and the resulting ability to colonise or infect the host 44, 45. Finally, polysaccharide antigens are more robust than protein antigens because biosynthesis loci do not include housekeeping genes that are required for other functions, and monosaccharides – unlike proteins – do not have specific binding functions which can be disrupted by single amino acid changes. Thus, polysaccharides are much more flexible than proteins in their changing ability, and modifications to their structure are unlikely to incur a major fitness cost to the organism. This is evident from successful persistence of isolates that have lost the capsule 46, 47, or loss of polysaccharide antigens in historical, *in vitro* passaged bacterial isolates 4, 48.

The evidence therefore points to polysaccharide antigen loci having universally evolved as unique bacterial adaptive weapons, able to diversify at different speeds [49]. Over short timescales, diversity can be produced within populations via regulation and phase variation; over intermediate timescales available serotypes are exchanged between lineages via recombinational events spanning entire loci; over long timescales, gene loss and gene gain via horizontal gene transfer produce novel, polymer-specific combinations, giving rise to new serotypes. Polysaccharide antigen loci thus allow rapid bacterial adaptation to constantly changing selective pressures, thereby maintaining fitness relative to other competing strains or species.

## Factors Shaping the Diversity of Polysaccharide Antigens

The genetic and phenotypic plasticity of polysaccharide biosynthesis loci can give rise to many serological types within a single species (Table 1), but it is not obvious why such diversity arose in the first place. Arguably, the simplest explanation is that diversity is randomly generated over time, but the drift alone is unlikely to be the driving evolutionary force. The sheer diversity of polysaccharide antigens in some species – much greater than expected based on the rest of the genome 4, 6, 50 – undoubtedly suggests the role of diversifying selection in generating new structures. The existence of such selection is also supported by the fact that polysaccharide synthesis loci are genetic variability hotspots between close bacterial species in the human gut [51], and they are often recombination hotspots, for which we have direct 41, 42, 52, 53, 54, 55, 56, 57 and indirect evidence 4, 35, 58, 59, 60, 61. Finally, bacterial polysaccharides often involve synthesis of rare sugars, like l-rhamnose or l-fucose, which are not typically found in animal cells [62]. This suggests that the benefit of the possibility of generating a greater number of polysaccharide combinations overcomes the cost of being more visible to the immune system. Therefore, it is now widely accepted that polysaccharide antigens can be, and often are, under strong diversifying selection.

**Figure 2:**
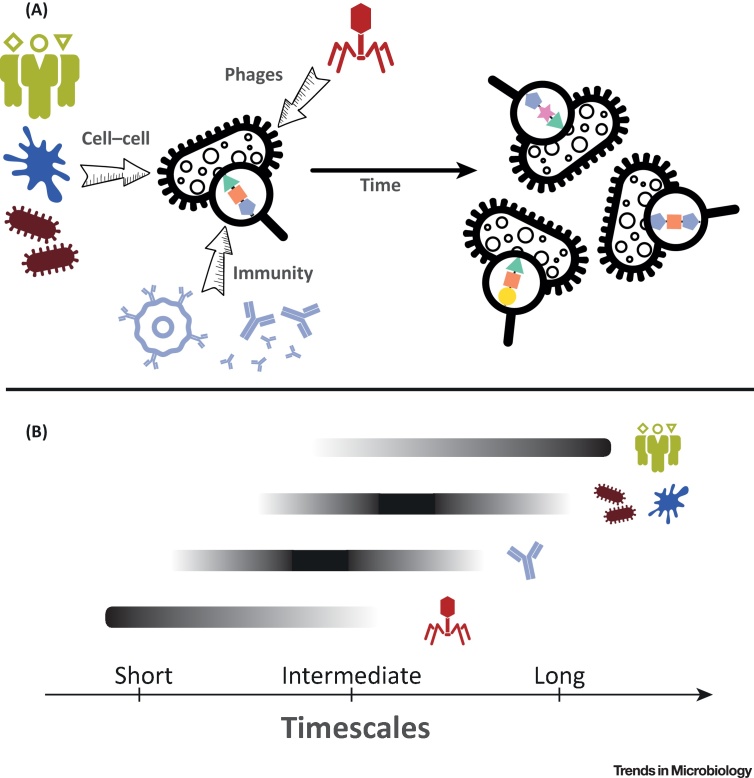
Factors Driving and Maintaining Diversity of Polysaccharide Antigens. (A) Major diversifying forces in the world of bacterial polysaccharide antigens: host immunity, bacteriophages, and cell–cell interactions (including host glycan diversity, eukaryotic predators and other host commensals). These forces are likely to drive and maintain the polysaccharide antigen diversity we observe today. (B) These factors should not be viewed as mutually exclusive, but rather as different forces operating at different scales of time and space. Coevolution with bacteriophages could select for novel polysaccharide diversity on short timescales from just a few bacterial generations, could occur within virtually any ecological niche including host associated or non-host associated, and could have transient or long-lasting impacts on both bacterial and phage population dynamics. The impact of genetic variation in host glycan diversity is expected to take much longer to affect bacterial population structures, depending on host diversity and generation times. Host immunity, the diversity of other host commensals, and the impact of predators are likely to operate somewhere between the two. Phages may promote within-host diversity of antigens, but a serotype which provides resistance against a given phage population may not spread in the population due to its low between-host fitness. Likewise, glycan diversity in different host populations may promote diversity over space: different populations found in different locations may promote different bacterial antigens, but a single type within each population.

The strength of diversifying selection will depend on the species ecology, which can be subdivided into three different classes of ecological factors: host immunity, bacteriophages, and cell–cell interactions (Figure 2). Importantly, each of these factors contains a coevolutionary component in that each involves interactions with other evolving entities. The strength of selection will also vary with the antigen type, and the biological role and interactions of that antigen are context-specific in each organism. For example, *K. pneumoniae* expresses both K- and O-antigens, but exhibits far more capsular variation than LPS variation; whereas the closely related enterobacterial species *Salmonella enterica* (mostly unencapsulated) and *E. coli* (variably capsulated) display extensive LPS diversity. However, in general, our understanding of the relative importance of the three ecological forces for bacterial evolution and the resulting antigenic diversity is far from complete.

### Host Immunity

Both capsules and O-antigens are known to interact with the immune system and can be immunogenic 14, 63. Correspondingly, host immunity has been the most popular candidate for diversifying selection of polysaccharide antigens in host-adapted bacteria. A classic model of immune-driven diversification is via negative frequency-dependent selection (Red Queen). Under this model, a new antigenic variant is under positive selection as it encounters naive hosts, but over time hosts develop immune memory against it and it is replaced by another, novel variant. This cycle continues, thus promoting antigenic diversity. However, under this scenario one would expect that novel diversity is constantly generated and dynamically changes over time. It has been argued that this model might explain the ability of some bacteria to alter their antigens on a generation timescale (like LPS variation in *N. meningitidis* and *Haemophilus influenzae*, or variable capsule expression in *Bacteroides*), which may promote colonisation and transmission by escaping the immune system [64]. But in many species the Red Queen model does not fit empirically observed temporal and spatial timescales at which novel serotypes emerge in many bacteria [65]. This conundrum has been partially resolved by mathematical modelling, which has by now quite convincingly demonstrated that immunity can play an important role in shaping the population structure of some pathogens and help maintain their antigenic diversity [66]. One class of multistrain models suggests that strain-specific immunity forces pathogens competing for hosts via cross-reactive responses to organise into non-overlapping antigenic repertoires [67]. It was argued that this framework explains the population structure of *N. meningitidis* and *S. pneumoniae* 68, 69. Furthermore, a balance between strain-specific immunity in naive infants (stabilising selection) and nonspecific immunity in adults (equalising selection) was suggested to explain the observed patterns of serotype diversity in the pneumococcus [70].

Nevertheless, immune-driven diversification of polysaccharide antigens is probably the exception rather than the rule in the bacterial kingdom. A recent study has shown that capsules are more common in environmental than in pathogenic bacteria, and also in facultative than in obligate pathogens [71]. The authors have argued that, for this reason, capsules have evolved as tools for environmental adaptation rather than as host-associated virulence factors. This is also consistent with the fact that, amongst all discussed bacterial species, the most extensive capsular variation is found in gut microbes that behave as opportunistic pathogens, such as *K. pneumoniae* and *E. coli*. Furthermore, extensive capsule diversity is also found in commensal bacteria (e.g., oral and pharyngeal streptococci [60], some gut microbiota species [51]), which are not affected by antibodies or phagocytes due to immunological tolerance to mutualistic members of our microbiome. All of this suggests that, while immunity can play a role in shaping polysaccharide antigen diversity, it cannot be the single and most important factor driving such diversity in all bacteria.

### Bacteriophages

Another factor that can act as an important driver of antigen polysaccharide diversity is bacterial coevolution with their viruses, namely bacteriophages (phages). Phages often encounter physical barriers preventing them from infecting bacterial cells: capsules, peptidoglycans, or extracellular polysaccharides in biofilms. Thus, phages have evolved strategies to overcome these barriers, and often carry receptors containing depolymerase – an enzyme which degrades polymers like extracellular polysaccharides present on bacterial surfaces. These receptors are typically encoded in tail fibres or base plates, and are highly specific to the antigen type. Consequently, diverse serotype-specific depolymerases have been identified in phages isolated from many different bacterial species [72]. Microbiological experiments have demonstrated that, over time, antagonistic coevolution between bacteria and their viruses will lead to an increased diversity of both bacteria and phages over time 73, 74. This hypothesis is in line with the dynamics observed in longitudinal analyses of the gut microbiome, which have shown dynamic shifts in bacterial and phage communities over time [75] and capsular polysaccharides as variability hotspots [51]. Temperate phages could also play a role in polysaccharide antigen diversification. They are known to coinfect bacteria [76], undergo competition 77, 78, and alter the chemical composition of O-antigen upon entry to prevent other phages from coinfecting, which has been well documented in several species 79, 80, 81.

### Cell–Cell Interactions

Cells are surrounded by carbohydrate structures known as glycans, and interaction between those glycans and proteins of other cells is expected to be an important ecological factor shaping their diversity [82]. Given the plethora of different cell–cell interactions in nature, we here focus on three important types of interaction which are central to the survival of bacterial pathogens and commensals inside hosts: interactions with host cells, with other colonising bacteria, and with predatory eukaryotes.

Host tissues, such as gut or respiratory epithelia (but also plant cell walls), are covered by a dense layer of glycans called the glycocalyx, which impacts many colonising bacteria. Consequently, microbes have evolved various ways of interacting with these structures to facilitate colonisation, including attachment to host glycans [82], modification of host glycans 83, 84, and biofilm formation [85]. As these interactions are necessary for successful colonisation, bacteria have been under evolutionary pressure to adapt to the spectrum of glycans expressed by the cells of their hosts. One consequence of this is the phenomenon of ‘molecular mimicry’, whereby some bacterial polysaccharide antigens have evolved to resemble the glycan structures of their hosts [86]. Such resemblance allows the bacteria to take advantage of self-tolerance in order to evade the host immune system. Examples include production of polysialic acid in *N. meningitidis* serogroup B, chondroitin or heparosan in *E. coli*, hyaluronic acid in some species of *Streptococcus* (including *S. pyogenes*, *S. equi*, *S. dysgalactiae*, and *S. uberis*) [86], or blood-group-antigen-resembling LPS in *Helicobacter pylori* or *Campylobacter jejuni* 87, 88. These adaptations are part of a larger coevolutionary dynamic between hosts and bacteria, whereby hosts diversify their glycans over time to escape the selective pressure of bacteria, which in turn coadapt (for example via molecular mimicry) [89]. In vertebrates, such diversification can also rely on somatic mechanisms (like hypermutation or recombination), which permit keeping up with rapidly evolving prokaryotes [82]. All of this generates not just large between-host diversity of glycans, but also diversity within individual hosts. Such diversity can be beneficial by inhibiting the spread of pathogens within a host population, due to the pathogen’s differential recognition of distinct glycan structures expressed by members of the host population [82].

In addition to host cells, bacteria colonising new hosts may encounter other prokaryotic and eukaryotic cells sharing the same niche. First, successful colonisation requires compatibility with host-associated commensal bacteria – for example, the mutualistic streptococcal communities found on oral mucosal surfaces. These communities are shaped by interactions between lectin-like adhesins and polysaccharides which act like receptors, thus connecting multiple cell–cell adhesions into large complex networks known as biofilms [90]. Since joining a biofilm substantially increases the chances of survival, new colonisers will be under selective pressure to attach and join the biofilm, or perish. This, in turn, will affect the population diversity of spreading bacteria [90]. Second, other single-celled species that feed on bacteria may also be present. Well known examples are the predatory eukaryotes, such as amoebae, which are sometimes found in animal intestines. As demonstrated in *S. enterica*, these predators exhibit different feeding preferences towards different O-antigens [91]. The specificity of such recognitions between a bacterivorous amoeba, *Acanthamoeba castellanii*, and *E. coli* has been attributed to the interaction between LPS and the predator’s surface mannose-binding protein [92]. Given the genetically characterised diversity of mannose N-glycans between different species of amoebae [93], it is plausible that such interactions have shaped (at least partly) the diversity of O-antigens in some bacteria, as has been argued previously [64].

## Selection over Time and Space

A schematic summary of the factors discussed above, and their expected timescales, is given in Figure 2. Importantly, one needs to be cautious when attempting to find a single explanation for the diversity of polysaccharide antigens in a given bacterial species, as forces maintaining it might be different from those which have driven it in the first place. For example, in *S. pneumoniae* it is widely accepted that immune selection is an important driver of antigenic population structure. However, it is not entirely clear whether occasionally appearing capsule switches are evolutionary ‘mistakes’, or whether they are (at least partly) driven by interactions with bacteriophages. Pneumococcal capsule-specific depolymerase enzymes have been isolated before 94, 95, but almost nothing is known about capsule–bacteriophage interactions in *S. pneumoniae*. In *Klebsiella*, some K-antigens are known to interact with the adaptive immune system [96], but frequent capsule switches in clinical lineages are unlikely to be explained by host immunity alone, and interaction with phages and protists are considered to be important [4], not least because these bacteria are found in the guts of many animals. It is also possible that, for a facultative pathogen like *Klebsiella*, the ability to exchange K-antigens permits adaptation during environmental shifts. In *S. enterica*, host-specific distribution of O-antigens was previously argued to be a consequence of the the varying feeding preferences of intestinal predators present in different animal hosts [91]. However, it has also been speculated that such diversity could be (at least partly) driven by the diversity of mucins (intestinal polysaccharides) which amoebae use for attachment: O-antigens resembling these attachments would be much less likely to get predated, thus providing bacteria carrying those polysaccharides with a selective advantage [97]. All of this emphasises how little we know about the relative importance of various ecological and evolutionary forces interacting with bacterial polysaccharide antigens. Their characterisation will be important for an accurate prediction of the short-term and long-term impact of therapeutic interventions (see also Box 2).

Box 2 **Predicting the Impact of Medical Interventions with Mathematical Models**

Mathematical models serve as powerful tools for predicting the effects of medical interventions. For a model to be a successful prediction tool, one needs to choose the right balance between the model simplicity and its biological accuracy.

**Polysaccharide Conjugate Vaccines**

Since the development and introduction of the conjugate vaccination programmes against *H. influenzae* and *S. pneumoniae*, mathematical models have been widely used to predict their effect on the control of bacterial disease 109, 110, 111. Such models are based on compartmental modelling, in which the human population is represented by different classes (for example vaccinated and unvaccinated), and rates of changes between these compartments are defined by a set of mathematical equations. Disease dynamics can be described deterministically (via ordinary differential equations) or stochastically (via parameters drawn from random distributions). These models have varying levels of complexity, depending on the underlying assumptions about the complexity of the host structure, pathogen biology, and interactions between the two.

Studies modelling the impact of conjugate vaccines have mostly focused on their short-term effects, namely their effect on bacterial transmission and disease. Some papers have examined the impact of conjugate vaccines on the bacterial population structure [112]; however, none have investigated the role of emergence of novel bacterial diversity on long-term vaccine effectiveness. This is difficult and requires models which can merge the epidemiological framework with pathogen genetics, as has been done with some viral disease models such as Influenza or HIV-1 113, 114. The predictive value of such models will depend not only on accurate estimates of epidemiological parameters (e.g., transmission rates, between-strain competition, strength of strain-specific and nonspecific immunity) but will also rely on good estimates of evolutionary rates driving diversification of bacterial genomes, including (but not limited to) acquisition of novel diversity from nonclinical reservoirs.

**Phage Therapy**

While phage therapy is fundamentally different from vaccination, previous studies have mostly relied on epidemiological models to predict its dynamics, and thus likely success 99, 115. However, these models still do not accurately capture the complexity of *in vivo* dynamics [116], nor do they address the question of coevolution between bacteria and viruses, or their potential epidemiological implications (e.g., transmission of bacterial resistance). Using mathematical models to predict the long-term success of phage therapy thus relies on quantifying parameters of *in vivo* bacteria–phage coevolution. Such quantification will be facilitated by recent advances in microbiome research and genomics [98].

Alt-text: Box 2

## Medical Implications

The adaptive potential of polysaccharide antigen loci to spread within bacterial populations or to evolve new types has potentially important consequences for public health. The antibiotic resistance crisis has led to an increased interest in polysaccharide-based therapeutic interventions (e.g., polysaccharide conjugate vaccines or phage-based therapies). These approaches impose strong selective pressures on a fraction of the bacterial population by targeting only a small subset of the capsular repertoire. Given the discussed potential of polysaccharide antigens to rapidly generate novel diversity, a question arises about the long-term efficacy of such interventions (see Outstanding Questions). We have seen some evidence of vaccine-driven adaptation in the antigenically diverse *S. pneumoniae*, which emphasises the challenge of robust vaccine design against other diverse bacteria like *Klebsiella* or *Acinetobacter* (Box 3). However, predicting the consequences of phage therapy will be arduous as current understanding of bacteria–phage coevolution in the complex environment of the human gut remains limited [98]. Also, since phage therapy is currently under active research and development, it is unclear what approaches will end up being used in practice. Using viruses as a public health control strategy has a long history of controversy, and indeed the implications of using bacteriophages for medical purposes have been actively debated for many years [99]. The rapidity with which some phage can kill their target suggests that the targeted bacteria would have a very narrow window of opportunity to evolve escape, for example by changes in the capsule locus; however, evolution experiments and observational microbiome data show that this may be possible or even likely 98, 100. One alternative might be to use phage-derived depolymerases which degrade bacterial capsules [101]; they alone would not kill bacteria and thus would impose very different selective pressures compared to lytic treatments.

Box 3 **Implications of Polysaccharide Diversity for Treatment Design: Lessons from Conjugate Vaccines**

While the significance of natural polysaccharide variation for vaccine escape has been appreciated for decades, recent advances in genomic epidemiology have improved our understanding about its actual importance. Our knowledge comes predominantly from vaccines against three nasopharyngeal pathogens: *Haemophilus influenzae*, *Streptococcus pneumoniae* and *Neisseria meningitidis*, the impact of which has been studied for many years.

The simplest form of vaccine escape is by losing the capsule, which is an acceptable outcome since unencapsulated bacteria are unlikely to cause disease. This is most likely to happen when a vaccine spans the entire capsule repertoire (as in cases where a single capsule serotype is associated with human-adapted invasive disease, such as in *Salmonella enterica* serovar Typhi or *H. influenzae* type B), but in fact in all three species there has been a rise in the reported frequency of unencapsulated strains 47, 117, 118. By contrast, when the vaccine spans only a fraction of the capsule repertoire, from an evolutionary point of view it becomes an ecological experiment where we can observe the impact of the removal of selected serotypes on the bacterial population (see also Figure IA). Interestingly, recombination-driven vaccine escapes have only been reported in the species which, incidentally, has the highest capsule diversity: the pneumococcus [119]. There, we have seen persistence of some lineages associated with vaccine serotypes thanks to exchange of those serotypes into nonvaccine serotypes via recombination 39, 54. Genetic sequencing from densely sampled areas also revealed the existence of previously unseen capsule loci that were recombinations of other serotypes [56]. Importantly, phylogenetic and phylodynamic analyses revealed that these recombinants emerged prior to the introduction of the vaccine 39, 54, implying that the polysaccharide capsule diversity evolves over time and that selection acts on this diversity. This process could be enhanced by interactions with closely related commensal bacteria, as we have seen that capsule genes are often shared between pathogens and commensals in all three bacterial species 59, 60, 61. Furthermore, in the pneumococcus there is evidence that commensals can act as a source of horizontally acquired capsule diversity 56, 120. It thus seems that the dynamic microbial ecosystem of the nasopharynx could be an evolutionary hub where novel serotypes occasionally emerge and, in the presence of a vaccine, gain a selective advantage and rise in frequency (Figure IB). However, as seen in the case of the three major pathogens, sharing a similar ecological niche, it is difficult to gauge the likelihood of such a scenario due to our limited understanding of the complex microbial interactions in the nasopharynx and the probability of new serotypes rising in frequency (see Outstanding Questions).

It will be even more challenging to predict the impact that polysaccharide conjugate vaccines in humans would have on populations of opportunistic pathogens with very different host ranges and ecologies, like *Klebsiella* or *Acinetobacter*. However, vaccines against these bacteria would most likely be used in a much more targeted way, such as aiming to protect at-risk patients in hospitals that are experiencing problems with outbreaks of antibiotic-resistant strains, or patients who are known to be colonised with such strains. Ultimately, estimating such risks requires a good understanding of the bacterial colonisation dynamics, which, in turn, emphasises the importance of routine bacterial carriage studies and quantifying bacterial evolution in real time.

Alt-text: Box 3

**Figure I:**
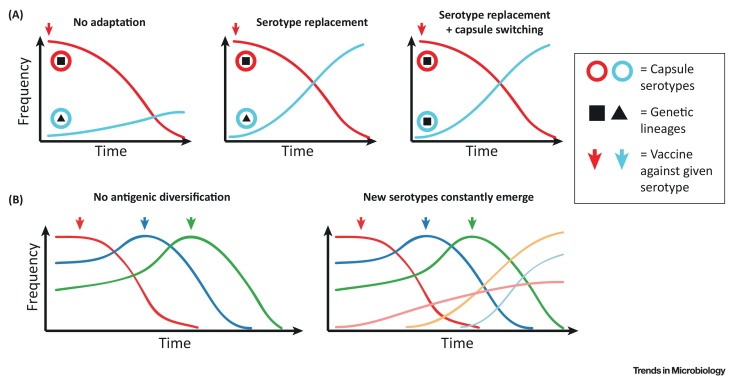
Mechanisms of Bacterial Adaptation against Polysaccharide Conjugate Vaccines. (A) Impact of polysaccharide conjugate vaccines on bacterial population structure. On the left, introduction of the vaccine against the red serotype is followed by the decline of this serotype but no replacement by another strain with the blue serotype, and thus overall reduction in carriage rates. This is similar to the situation in Haemophilus influenzae [121]. In the middle, vaccination against the red serotype is followed by the rise of another lineage (triangle) with the blue serotype with no significant reduction in carriage, known as ‘serotype replacement’. On the right, vaccination against the red serotype is followed the rise of the same lineage (square) with another serotype (blue). This is a result of an acquisition of the blue serotype by the square lineage (known as ‘serotype switching’), which had occurred prior to the introduction of the vaccine. The latter two situations are frequently observed in *Streptococcus pneumoniae*. (B) Potential impact of antigenic diversification on multivalent vaccine strategies. On the left, it is assumed that serotypes do not diversify over time. In this theoretical scenario, broader, multivalent vaccines could eventually lead to the eradication of the bacterial disease. On the right, new serotypes constantly emerge at low frequencies and are selected for by the vaccine due to serotype replacement. This scenario represents a Red Queen race between the vaccines and bacteria. In such a case, broader vaccines could select for novel, previously unseen serotypes to rise in frequency.

Ultimately, long-term effectiveness of any polysaccharide-targeted medical intervention will depend on a good understanding of the evolutionary dynamics of the relevant bacterial system, as well as on clever formulation of mathematical models to predict the impact of such approaches (see also Box 2). However, neither can be achieved without viewing bacterial pathogens in the wider context of their ecological interactions as such pathogens in reality represent a ‘tip of the iceberg’ of the entire bacterial population. As medical interventions – like antibiotics, vaccines, or phage therapies – will impact the bacterial ecosystem as a whole, more attention in the future should be devoted to isolate sampling designs that overcome such clinical bias. Fortunately, with the advancement of next-generation sequencing such studies are increasingly likely to become routine in the future.

## Concluding Remarks

Polysaccharide antigens, such as capsules, O-antigens, or teichoic acids, are common in pathogenic and commensal bacteria. Our understanding of how these structures are synthesised and how they interact with hosts, viruses, and the environment have largely improved over the last two decades. Altogether, they paint a picture of genetic loci which are highly adapted to become modified and exchanged between different bacteria. Such diversity is likely a result of millions of years of coevolution with bacterial viruses, hosts, predators, and other bacteria, and is unlikely to change drastically on epidemiological timescales. However, the introduction of vaccines, antibiotics, or phage therapies may dramatically alter the structure of bacterial populations due to the resulting strong selective pressures. It is thus conceivable that these antigens could rapidly evolve, undermining the long-term efficacy of therapeutic interventions. Therefore, the ability to predict the long-term consequences of these interventions and inform public health will be conditional on our understanding of the biology, epidemiology, and ecology of the system in question, quantification of evolutionary and epidemiological parameters, and the resulting accurate design of mathematical models.

Outstanding Questions What is the impact of within-host evolutionary dynamics on bacterial population structure? We know relatively little about within-host forces driving antigenic diversification, and about trade-offs between within-host and between-host bacterial fitness and transmission.

How important are polysaccharides in carriage and disease? Some capsule types are strong predictors of carriage and virulence, but their role in pathogenesis is not fully understood.

Can we efficiently predict phenotypes from genotypes? Genetic typing permits rapid identification of well known serotypes but it tells us little about the genotype–phenotype map.

How important are commensal and nonclinical strains in driving evolution of pathogens? Nonpathogenic bacteria are largely understudied, and we need to understand their role in pathogen evolution and diversification.

How fit are different serotypes in different environments? Environment-dependent measures of serotype fitness would help us predict the emergence of new serotypes in the future.

What impact do bacteriophages have on bacterial population dynamics? We need to understand how phages interact with bacteria and how they shape population structures of bacterial populations.

How common are interactions between eukaryotic predators and bacteria? We need more studies highlighting the nature of interactions between protists, bacteria, and their ecology, and need to understand how they impact evolution of non-host-associated bacteria.

What is the impact of host glycan diversity on pathogen evolution? Glycan diversity may affect the structuring of bacterial populations, both within-host and between-host.
